# Do progenitors play dice?

**DOI:** 10.7554/eLife.54042

**Published:** 2020-01-17

**Authors:** Esther Klingler, Denis Jabaudon

**Affiliations:** 1 Department of Basic NeurosciencesUniversity of GenevaGenevaSwitzerland; 2 Clinic of NeurologyGeneva University HospitalsGenevaSwitzerland

**Keywords:** neocortex, neuron, cell fate, cell lineages, modelling, cortical development, Mouse

## Abstract

The wide range of cell types produced by single progenitors in the neocortex of mice may result from stochastic rather than deterministic processes.

**Related research article** Llorca A, Ciceri G, Beattie R, Wong FK, Diana G, Serafeimidou-Pouliou E, Fernández-Otero M, Streicher C, Arnold SJ, Meyer M, Hippenmeyer S, Maravall M, Marin O. 2019. A stochastic framework of neurogenesis underlies the assembly of neocortical cytoarchitecture. *eLife* **8**:e51381. doi: 10.7554/eLife.51381

Understanding how the many different cell types that make up an adult organism emerge from the successive divisions of a single cell is a central question in biology. In particular, given the numerous random interactions that happen between and within cells (Vogt, 2015; Symmons and Raj, 2016; Hiesinger and Hassan, 2018), how do these multiple cell types reproducibly organize themselves into a robust body structure?

Consider the neocortex, a region of the brain that is involved in higher-order functions such as cognition and language. The neocortex contains a large number of different types of nerve cells called neurons, which are organized into six distinct layers. Neurons within each layer express specific genes and have distinct patterns of connections, which are key features for establishing proper brain circuitry (Jabaudon, 2017).

As the embryo develops, neurons are generated from mother cells called progenitors located below the neocortex: deep-layer neurons are born first, followed by superficial-layer neurons, in an 'inside-out' pattern during neocortical development. Although molecularly distinct mature neocortical neurons have been identified within each of the six layers (Tasic et al., 2016), a corresponding set of molecularly diverse neocortical progenitors has not been found (Telley et al., 2019). Thus, it remains unclear how exactly distinct neuron types emerge from a seemingly more uniform pool of progenitor cells.

Now, in eLife, Oscar Marín from King’s College London and co-workers – including Alfredo Llorca as first author – report that stochastic, random processes may explain how single progenitors are able to generate a wide range of neuron types (Llorca et al., 2019). The team – which includes researchers at King's College, the IST in Austria, the University of Freiburg and the University of Sussex – used three different techniques to genetically label progenitor cells, and then mapped the lineages (i.e. the neocortical clone) produced by each progenitor, focusing on progenitors that started generating neurons early during neocortical development. Of the clones that Llorca et al. were able to characterize, ~80% had daughter neurons in both the deep and superficial layers of the neocortex, while only ~20% had daughter neurons restricted to one of these two tiers. The neurons generated from individual progenitors also showed various axonal projection patterns and molecular identities, which corresponded to their layer position.

While this type of analyses has been done previously (Gao et al., 2014), these results provide important and thorough cross-validations of experimentally challenging approaches. In addition, Llorca et al. went one step further and developed mathematical models that could emulate their biological observations. They found that a model containing two types of progenitor cell which could randomly generate diverse ranges of neuron types was the best fit for their experimental results. These computer simulations suggest that a limited number of progenitors with a stochastic neuronal output can in principle account for the diverse clone types observed in the adult neocortex (Figure 1).

**Figure 1.**
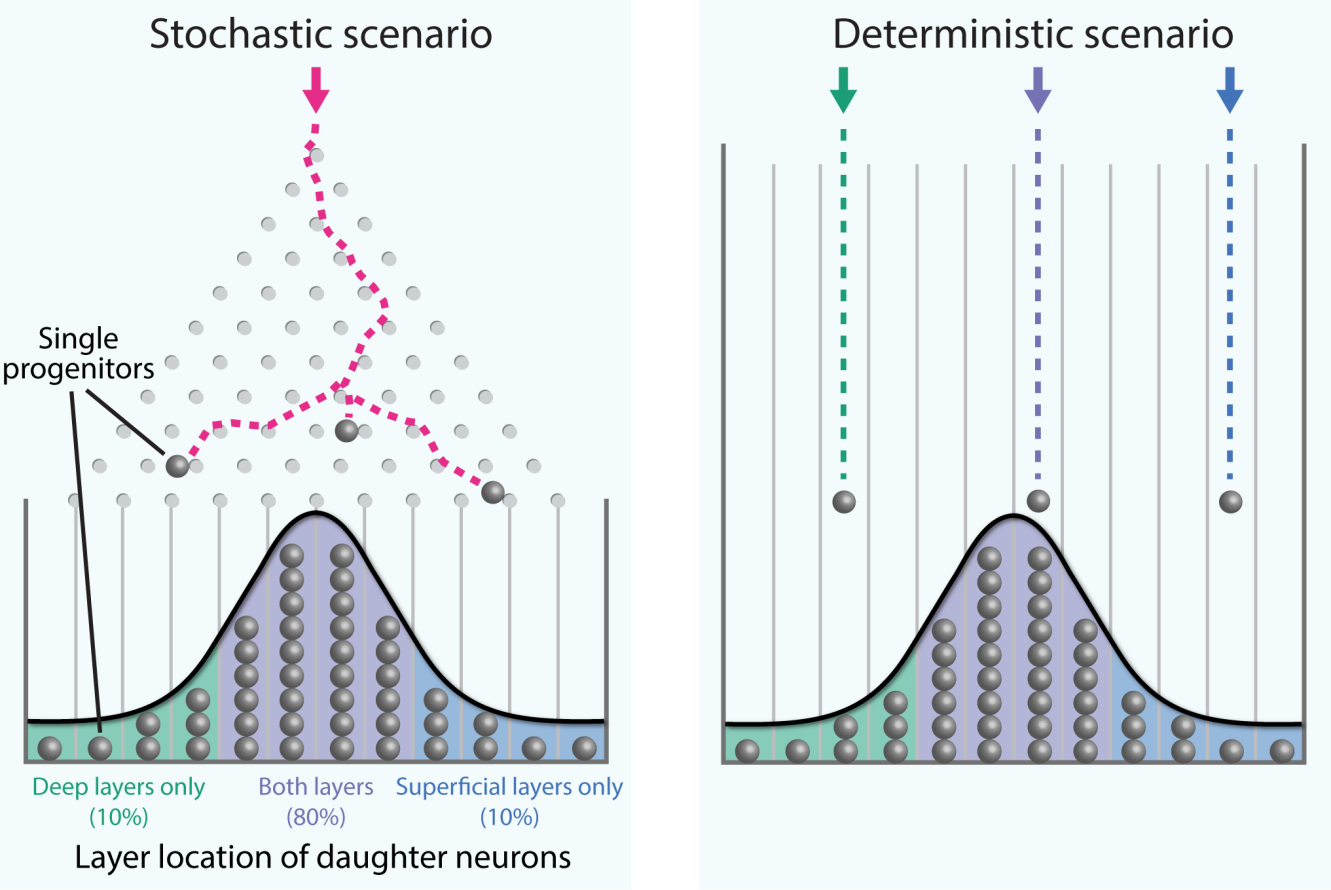
Stochastic generation of wide ranges of neuron types by single cortical progenitors. The path taken by a single marble as it rolls down an inclined plane through an array of pegs and into a row of bins (a device called a Galton board; see video for a live-action example) cannot be predicted (left). However, over time a distribution of marbles in the bins builds up, and when the number of marbles is large enough this distribution can be predicted. Llorca et al. propose that the diversity in the output of single progenitors in the mouse neocortex is best explained by a small number of progenitors undergoing such stochastic (i.e. random) fate choices, rather than each type of adult neuron developing in a deterministic manner from a corresponding fate-restricted progenitor (right). The distribution of progenitors within the bins reflects the experimentally observed laminar position of their daughter neurons.

Stochastic events are critical for other developmental processes (Vogt, 2015; Symmons and Raj, 2016; Hiesinger and Hassan, 2018), including the fate choices of blood cell progenitors (Chang et al., 2008). However, distinguishing genuinely stochastic processes from unidentified deterministic processes is difficult. While modern technologies are becoming more sensitive, their signal-to-noise ratio is not infinite, which means that complex deterministic processes involving multiple factors can appear to be stochastic.

Although the stochastic hypothesis of Llorca et al. is based on simulations, it is provocative and raises exciting questions that can be experimentally tested. For example, can the two progenitor subtypes proposed by the model be molecularly identified? Do stochastic events occur within progenitors themselves or in their neuronal progenies? And when it comes to shaping the fate of a neuronal cell, what are the relative contributions of stochastic and deterministic processes?

Most importantly perhaps, this study highlights the limits of a reductionist, cell-by-cell description of neocortical and embryo development. If dynamic cellular features are predictable at a population rather than single-cell level, developmental processes may be most efficiently addressed by understanding the emergent properties of populations of cells rather than by a detailed account of their individual components.
